# Finite element method for the design of implants for temporal hollowing

**DOI:** 10.1016/j.jpra.2021.12.001

**Published:** 2021-12-18

**Authors:** Federica Ruggiero, David Dunaway, Curtis Budden, Luke Smith, Noor Ul Owase Jeelani, Silvia Schievano, Juling Ong, Alessandro Borghi

**Affiliations:** a DIBINEM Alma Mater Studiorum University of Bologna, Bologna, Italia; b Craniofacial Unit, Great Ormond Street Hospital, London WC1N 1JH (UK); c Great Ormond Street Institute of Child Health, University College London, London WC1N 1EH (UK)

**Keywords:** Craniofacial surgery, Trigonocephaly, Temporal hollowing, Finite element modelling

## Abstract

Temporal indentations are the most impacting craniofacial complication after coronal flap dissection. It is mainly due to a temporal fat pad or temporalis muscle dissection.

Because of the great improvements achieved recently in CAD-CAM-aided surgery and the possibility of performing accurate pre-surgical virtual planning, it is now possible to correct it with a customised virtual approach. Furthermore, advancements in material science have allowed surgeons to rely on biocompatible materials like PEEK (showing a low complication and recurrence rate) for the manufacturing of patient-specific implants.

We hereby describe our experience on a case of secondary and corrective surgery after a fronto-orbital remodelling, in which we used PEEK implants designed by CAD and optimized by finite element modelling.

## Introduction

Coronal incision is one of the most used surgical approaches in major craniofacial surgery procedures. Temporal hollowing is one of the most frequent cosmetic complications after coronal incisions; although the aetiopathogenesis is still controversial, fat pad and temporalis muscle atrophy are thought to be the main causes.^1^^,^^2^

Since it is frequently a reason for cosmetic dissatisfaction and psychological distress for patients, several surgical methods have been developed to prevent it or treat it^2^^,^^3^

A wide range of secondary surgical solutions has been developed, ranging from autologous fat transfer to patient-specific implants (PSI) developed in biocompatible materials (polyether ether ketone (PEEK) and polymethylmethacrylate (PMMA)), because of the advent of technologies for CAD-CAM-aided surgery.^2^^,^^3^

We hereby present a case of temporal deformity due to previous fronto-orbital advancement remodelling (FOAR), where the patient (a 13-year-old boy) underwent a PEEK custom cranioplasty, planned by finite element modelling (FEM) prediction.

## Methodology and Results

**Figure 1:**
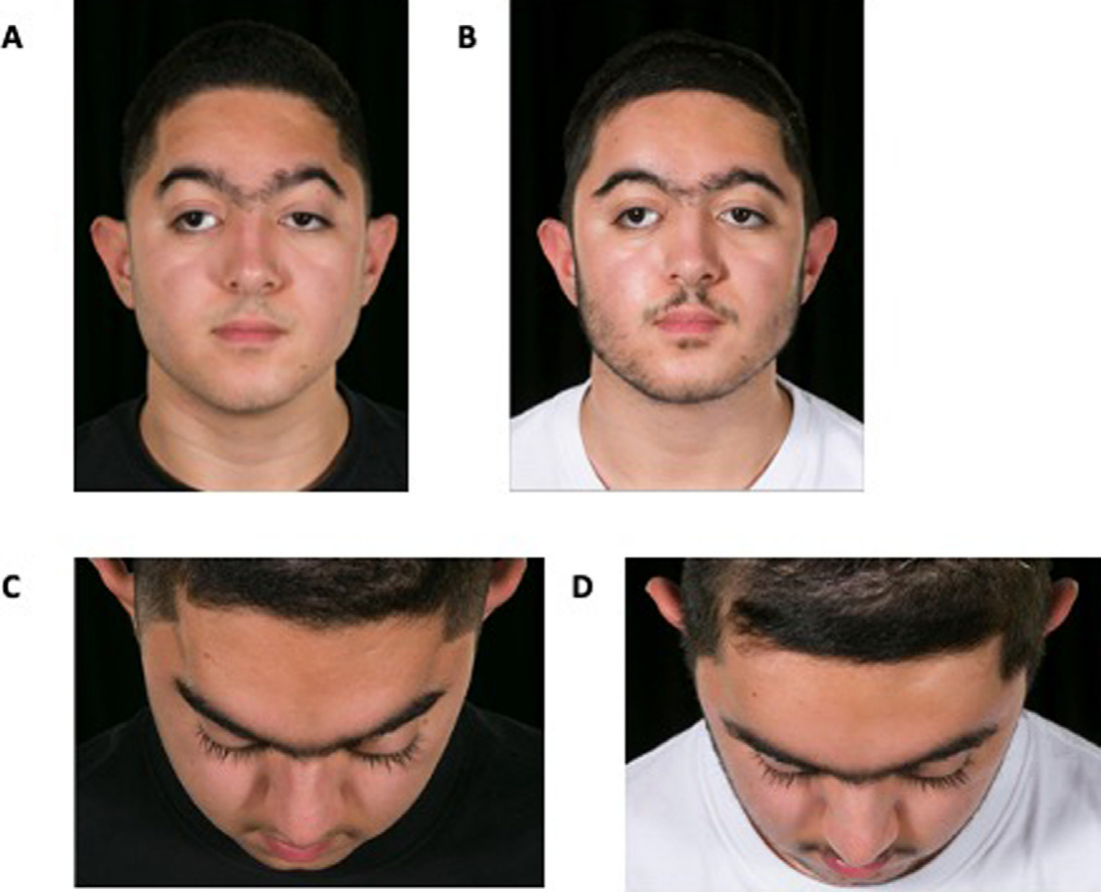
Patient's preoperative photos (A): temporal indentations may be noted in the frontal view. Postoperative photos (B): appearance correction achieved in the frontal view. Preoperative photo bird view (C). Postoperative photo bird view (D).

The patient was admitted to the Craniofacial Unit at Great Ormond Hospital for Children in London to undergo FOAR in 2005 at the age of 1 year following a diagnosis of metopic craniosynostosis. Twelve years later (at the age of 13), the patient noted indentations at the temporal regions (Figure 1A-C).

**Figure 2:**
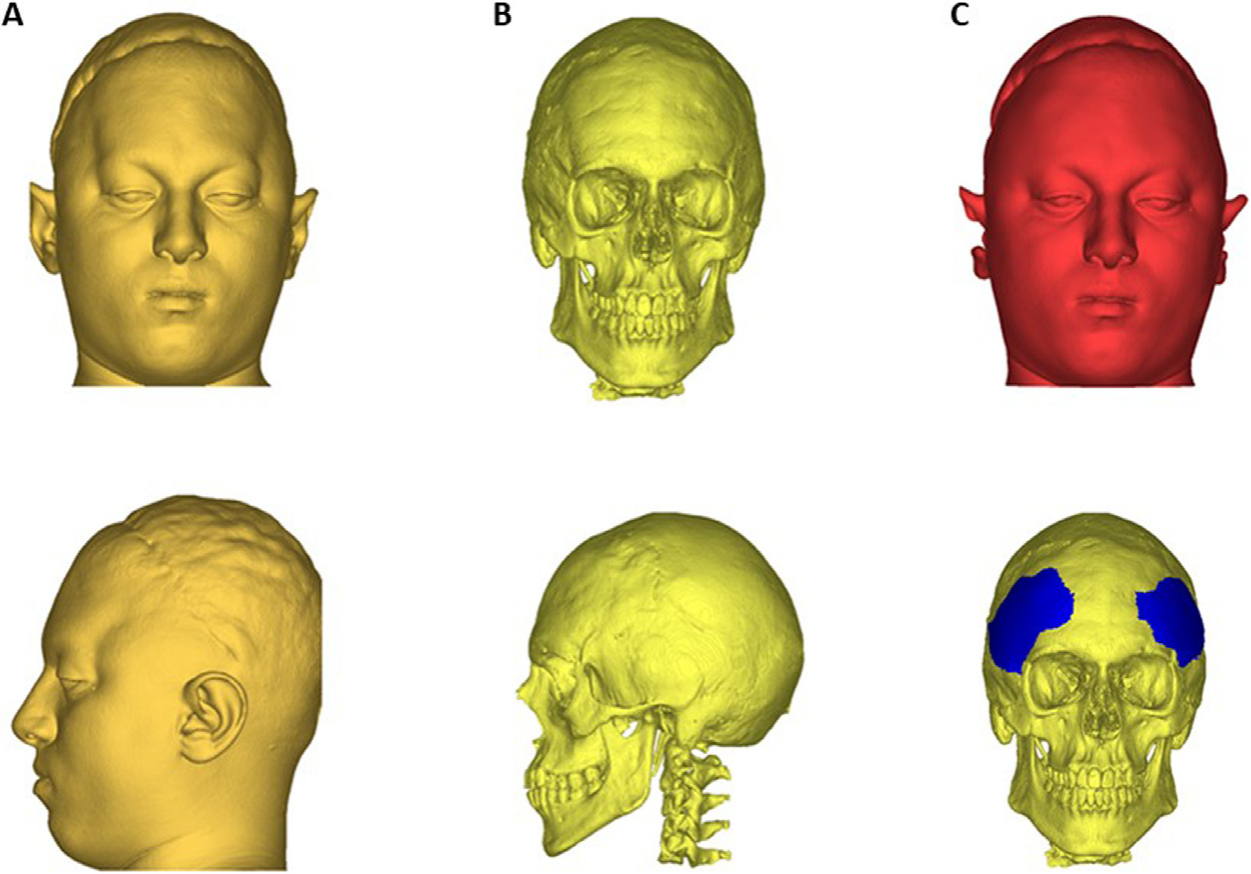
Preoperative assessment: (A) preoperative soft tissue reconstruction (top: frontal view, bottom: side view); (B) preoperative bone reconstruction (top: frontal view, bottom: side view); (C) reconstruction of the desired soft tissue (top) and hard tissue with implants overlaid (bottom)

A CT scan was performed for preoperative assessment of soft and bony tissue. The CT scans were imported into Mimics Innovation Suite (Materialise, Belgium) which was used to perform 3D soft and hard tissue reconstruction (Figure 2A-B). The soft tissue model was processed by an experienced plastic surgeon (DD) by using MESHMIXER (Autodesk, USA) to create a desired shape of the forehead (Figure 2C, top). The soft tissue was offset by a constant value (5.6 mm, equal to the most frequent value of soft tissue thickness in the area), and the skull volume was subtracted to obtain two volumetric patches (implants) in the region of the indentation, which were suitable for stretching the outer skin to gain the desired shape (Figure 2C, bottom).

**Figure 3:**
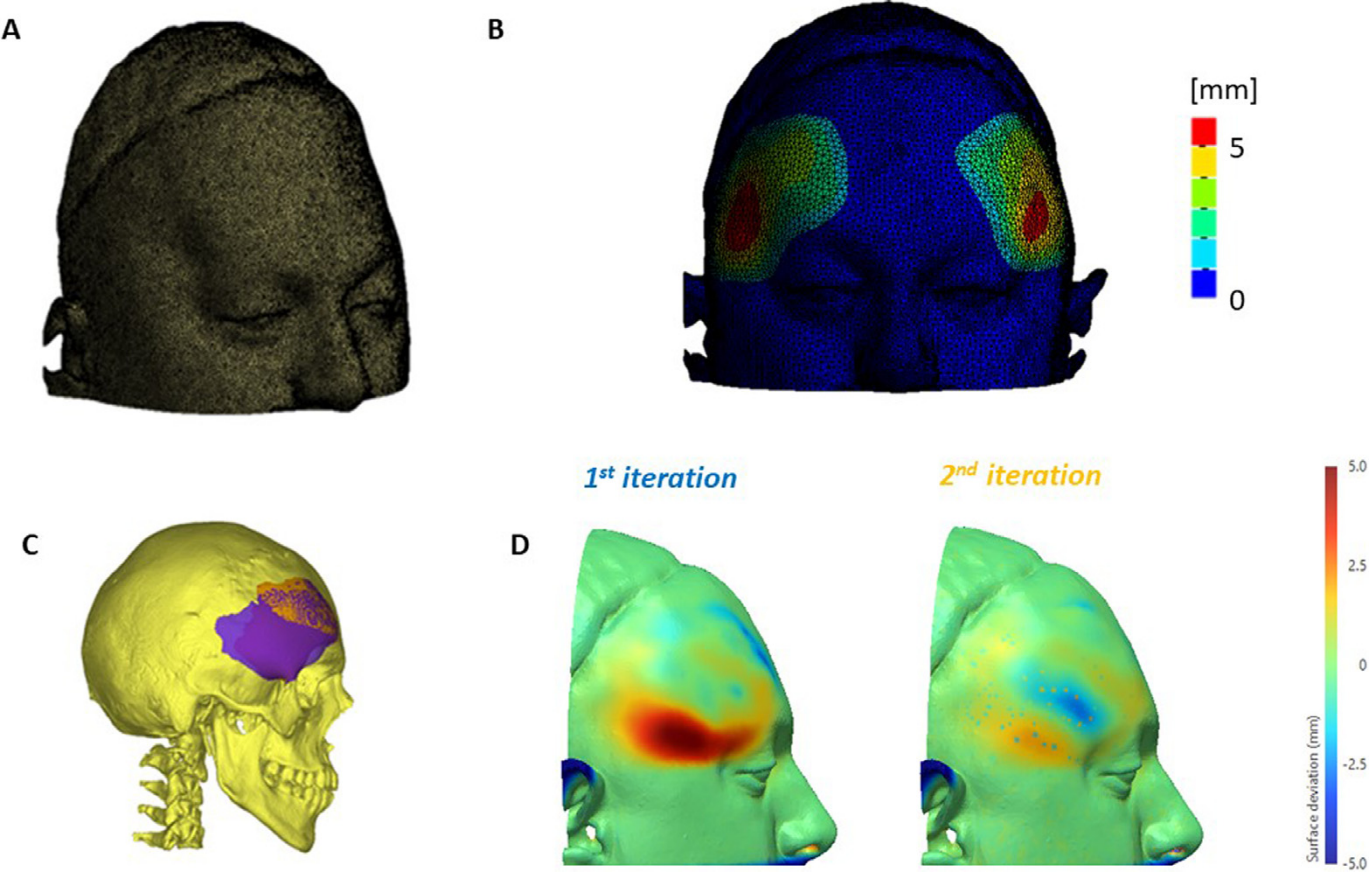
Preoperative implant optimization by finite element modelling: (A) discretisation of preoperative soft tissue reconstruction; (B) surface changes after simulated implants insertion; (C) visualization of different implant iterations, first (blue) and second (yellow); (D) comparison of simulation outcomes to the desired shape (figure 1C) in terms of surface distance, first iteration (left) and second iteration (right)

To test the effect of implant insertion, FEM (a numerical method to assess physical interaction between elastic bodies) was used. The upper face of the patient was discretized (Figure 3A) and imported in ANSYS Mechanical (ANSYS, USA); skull tethering was simulated by constraining the areas distal from the implants. The implants were inserted in the correct anatomical position, allowing the soft tissues to respond elastically (the soft tissue was modelled as elastic isotropic material with Young's modulus E = 1MPa and Poisson's ratio ν = 0.499).^4^

The simulations (Figure 3B) were compared with the desired outer soft tissue shape (Figure 3D), and the results showed that the implants were correctly shaped in the supraorbital region, while in the infraorbital region (where a non-distribution of the soft tissues was present), the amount of soft tissue change was higher than that required (Figure 3D, left). A second attempt was performed, where implants were manually modified in the lower part (Figure 3C). The results showed a better adherence to the desired final soft tissue shape (Figure 3D, right). The implants were then manufactured in PEEK by an external company (Cavendish, UK) after authorization of the patient's parents was obtained.

**Figure 4:**
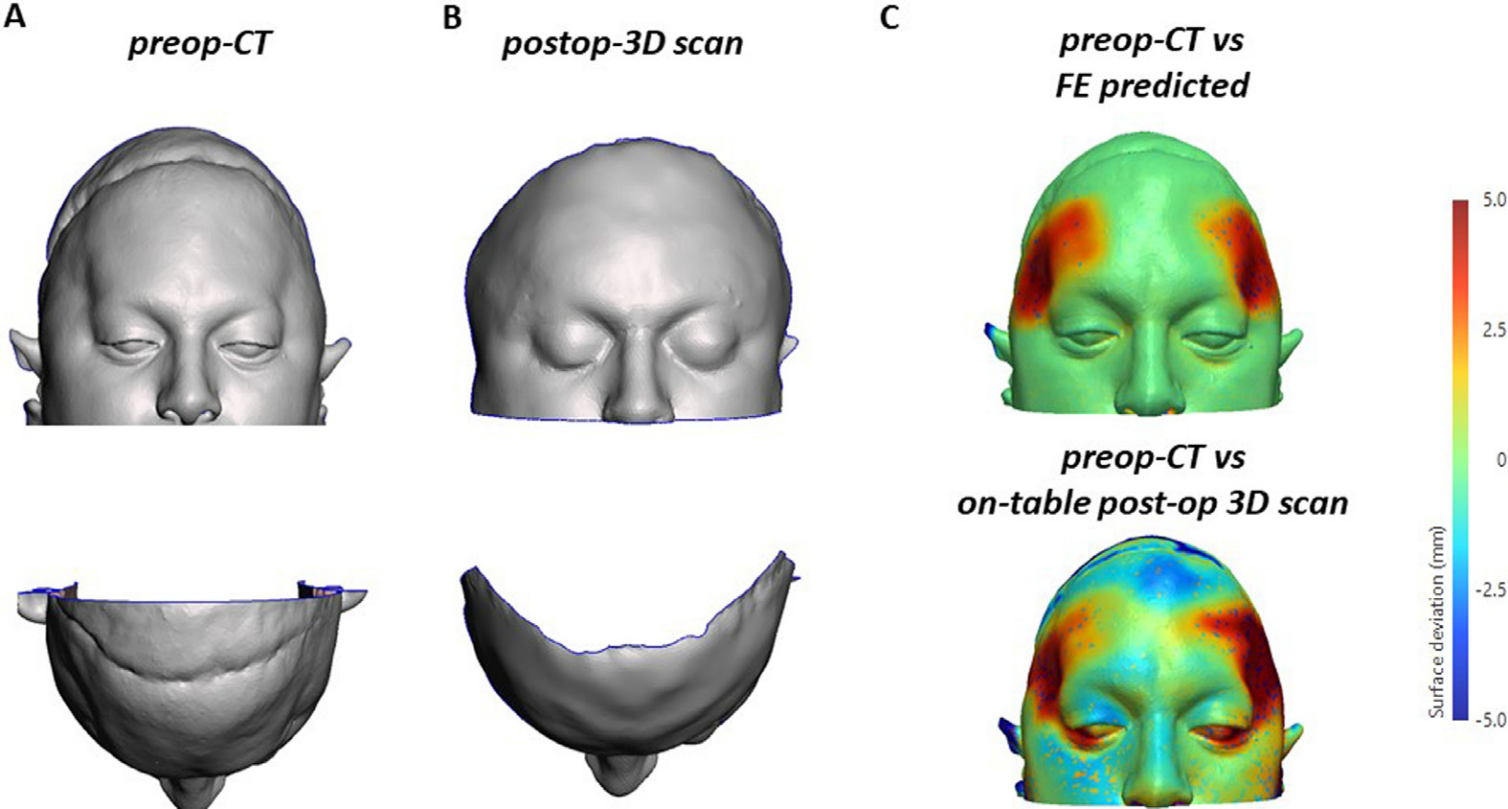
Postoperative outcomes: (A) preoperative CT scan 3D soft tissue reconstruction (top: frontal view, bottom: bird view); (B) postoperative 3D on table scan reconstruction (top: frontal view, bottom: bird view); (C) desired tissue reshaping (preoperative CT vs FE prediction, on top) versus postoperative surgical outcome (preoperative CT vs on-table post-op 3D scan) visualised in terms of surface distance.

The implants were positioned in the theatre. The pre-existing coronal incision was re-opened after injection of tumescent solution. The coronal composite flap was then raised in the sub-pericranial plane. The temporalis muscles appeared to be contracted and attached more inferiorly than normal on the skull. Afterwards, implants were secured to the cranium with titanium mini-screws, and the temporalis muscles were resuspended by pre-designed drill holes in the implant with 3-0 PDS suture. The entire surgical procedure lasted for 90 minutes. The patient was discharged on the first post-operative day after an uneventful recovery. The preoperative CT (Figure 4A) was qualitatively compared to a post-operative 3D scan that was retrieved on-table (Figure 4B). Surface difference colour maps were produced to compare the simulated soft tissue reshaping pattern (Figure 4C, top) with the one obtained after implant insertion (Figure 4C, bottom).

## Discussion and Conclusion

PEEK patient-specific implants have been successfully used in craniofacial surgery for over 20 years.^2^ Their popularity is because of due to the flexibility of the material in terms of biocompatibility, radiological compatibility and biochemical inertia.^5^

The findings in the literature are controversial when it comes to the complication rates of PEEK implants, but a few have been reported relatively to the use in the case of cranioplasty.^2^^,^^5^, ^6^, ^7^ In our unit experience, PEEK is a viable and reliable material with good long-term results in terms of stability and cosmetic appearance.

The numerical model used to design the implant is simplified and only includes soft and bony tissues, which were modelled as linear materials. This assumption may affect the accuracy of the simulation results in terms of stress analysis, but – as the aim of the simulation was to predict the soft tissue displacement – it bears no effect on the implant design pipeline hereby presented. This is also reflected in the good matching between the simulated tissue response and the actual surgical outcomes as shown in Figure 4C. We acknowledge that a more complex model of the patient upper face (including anatomical sub-structures such as muscles, mucosae and three layers of the skin)^8^ and patient-specific tissue mechanical properties^9^ would yield more accurate results.^10^ Nevertheless, the hereby simplified model provided acceptable results, and the FEM-designed PSI achieved the desired outcomes once implanted (Figure 1 B,D).

This case demonstrates the advantages of surgical planning of complex soft tissue augmentations using FEM to accurately design the shape and the position of the implants. Although PEEK is expensive, total expenditure was offset by the reduction in operating time and hospital stay. In our experience, PEEK PSIs have been a reliable technique to overcome the temporal hollowing.

## Declaration of Competing Interest

None
